# MAP kinase phosphatase 2 deficient mice develop attenuated experimental autoimmune encephalomyelitis through regulating dendritic cells and T cells

**DOI:** 10.1038/srep38999

**Published:** 2016-12-13

**Authors:** Mark Barbour, Robin Plevin, Hui-Rong Jiang

**Affiliations:** 1 Strathclyde Institute of Pharmacy and Biomedical Sciences, University of Strathclyde, 161 Cathedral Street, Glasgow, UK

## Abstract

Mitogen-activated protein kinase phosphatases (MKPs) play key roles in inflammation and immune mediated diseases. Here we investigated the mechanisms by which MKP-2 modulates central nervous system (CNS) inflammation in experimental autoimmune encephalomyelitis (EAE). Our results show that MKP-2 mRNA levels in the spinal cord and lymphoid organs of EAE mice were increased compared with naive controls, indicating an important role for MKP-2 in EAE development. Indeed, MKP-2^−/−^ mice developed reduced EAE severity, associated with diminished CNS immune cell infiltration, decreased proinflammatory cytokine production and reduced frequency of CD4^+^ and CD8^+^ T cells in spleens and lymph nodes. In addition, MKP-2^−/−^ CD11c^+^ dendritic cells (DCs) had reduced expression of MHC-II and CD40 compared with MKP-2^+/+^ mice. Subsequent experiments revealed that CD4^+^ T cells from naïve MKP-2^−/−^ mice had decreased cell proliferation and IL-2 and IL-17 production relative to wild type controls. Furthermore, co-culture experiments showed that bone marrow derived DCs of MKP-2^−/−^ mice had impaired capability in antigen presentation and T cell activation. While MKP-2 also modulates macrophage activation, our study suggests that MKP-2 is essential to the pathogenic response of EAE, and it acts mainly via regulating the important antigen presenting DC function and T cell activation.

Experimental autoimmune encephalomyelitis (EAE) is an inflammatory demyelinating disease of the central nervous system (CNS). It is the most commonly used animal model for the study of human multiple sclerosis (MS), a condition which affects approximately 2.5 million people worldwide and is one of the leading causes of neurological disability in young adults. EAE is thought to be mediated predominantly by effector Th1 and Th17 cells activated by antigen presenting cells, which leads to demyelinating CNS inflammation.

Mitogen-activated protein kinases (MAPKs) control a vast array of important physiopathological processes including various immune responses to stimuli/stress/damage in multicellular organisms. MAPK phosphatases (MKPs) are a group of dual specific phosphatases (DUSPs) which deactivate the MAPKs (i.e. ERK, JNK, p38) via dephosphorylation of phosphotyrosine and phosphothreonine residues, and thus play a key role in inflammation mediated diseases. Indeed various MKPs including MKP-1, MKP-5, MKP-7, MKP-x (DUSP22) and DUSP5 have been shown to be important in regulating immune responses^1^,^2^,^3^,^4^,^5^,^6^. For example, MKP-1 negatively regulates the production of inflammatory cytokines TNF-α, IL-6 and IL-1β, and the anti-inflammatory IL-10^7^,^8^,^9^, as well as chemokines and other inflammatory mediators^10^,^11^,^12^,^13^. Increased immune responses have also been observed in MKP-1 deficient mice after LPS administration compared with wild type controls^14^. During CNS inflammation, EAE severity is ameliorated in the absence of MKP-1^15^ and MKP-5^16^ while MKP-x deficient mice are more susceptible to EAE^17^.

MKP-2 is a dual-specificity phosphatase (DUSP-4) localised within the nucleus and is expressed in a wide range of cells and tissues including the CNS^18^,^19^. It regulates ERK, JNK or p38 pathways depending on cell type. MKP-2 is well documented to be an important immune response modulator in a number of diseases. In acute lung injury (ALI), MKP-2^−/−^ mice had reduced TNF-α and MIP-1α production and neutrophil lung infiltration^20^, while a significantly reduced mortality was also exhibited in the gene deficient mice in sepsis which was associated with decreased serum levels of TNF-α, IL-1β, IL-6 and IL-10^21^. We recently reported that MKP-2 deletion led to a greater susceptibility to *Leishmania Mexicana*^22^ and *Toxoplasma gondii*^23^ infection in mice, but with different underlying immune mechanisms.

The role of MKP-2 in CNS inflammatory diseases such as MS is currently unknown, this study investigated the function of MKP-2 during the development of CNS inflammation in EAE using MKP-2 deficient mice. Our data show that the mRNA expression levels of MKP-2 were significantly upregulated in the CNS and lymphoid organ tissues of EAE mice compared to naïve control animals. When EAE was induced in MKP-2^+/+^ and MKP-2^−/−^ mice, we observed reduced clinical scores of EAE in the gene deficient mice, which was associated with reduced inflammation and immune cell infiltration in the spinal cord tissues. Analysis of immune responses indicates that the frequency of CD4^+^ and CD8^+^ T cells, and CD11c^+^ MHC-II^+^ dendritic cells (DCs) in the peripheral lymphoid organs was decreased, together with reduced production of antigen specific pro-inflammatory cytokines by the lymphoid cells. Finally our experiments using bone marrow derived DCs (BMDCs) suggest that DCs from MKP-2^−/−^ KO mice had significantly reduced expression of MHC-II and costimulatory molecules and impaired capability in T cell activation, in addition to the intrinsic defect of MKP-2 deficient T cells in activation and cytokine production.

## Results

### Expression of MKP-2 mRNA is increased in tissues of EAE mice

**Figure 1:**
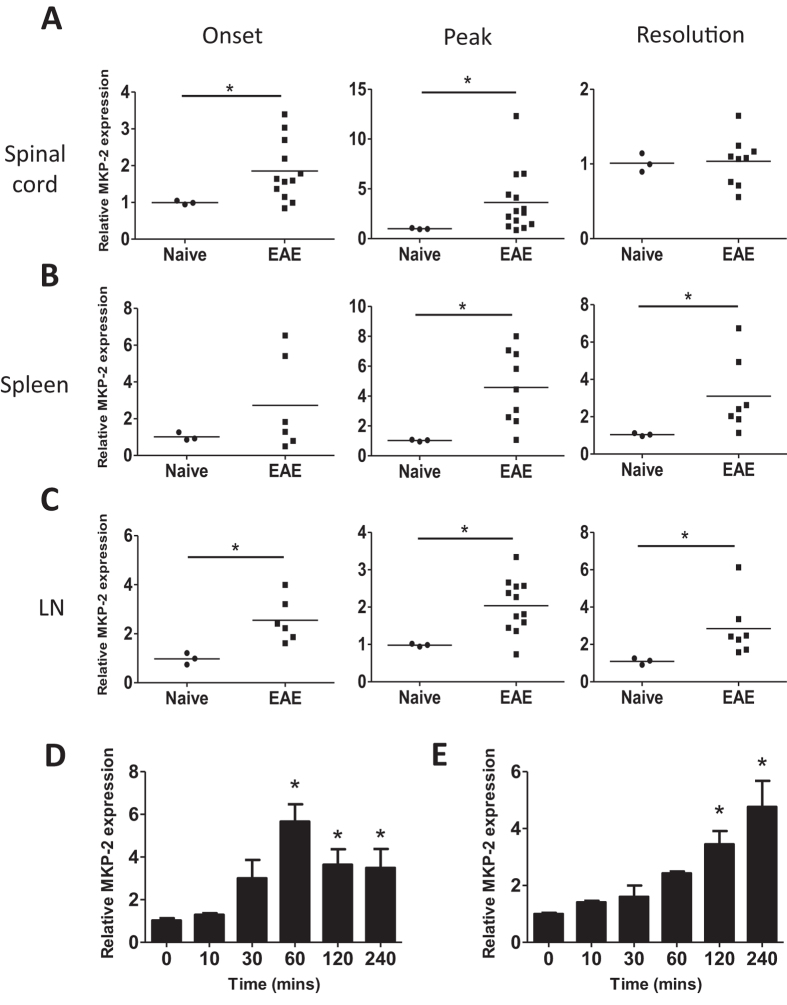
MKP-2 mRNA expression is increased in tissues of EAE mice. (**A**) Spinal cord, (**B**) spleen and (**C**) dLN tissues were harvested from naïve C57BL/6 mice or at EAE onset, peak and resolution stages of MOG_35-55_ immunised mice. RNA was isolated and reverse transcribed to allow analysis of MKP-2 mRNA expression by quantitative PCR. (**D**) Spleen and (**E**) dLN tissues were harvested from EAE peak mice and single cell suspensions prepared and cultured with or without MOG_35-55_ for the times indicated. Cells were then harvested for analysis of MKP-2 mRNA expression by quantitative PCR. Results show changes in MKP-2 mRNA expression relative to naïve tissue or unstimulated cells using the 2-ΔΔCT method. (**A–C**) Each symbol represents an individual mouse and bar indicates the average value of relative MKP-2 expression in each group. (**D–E**) Graphs show data from two independent experiments, n = 8. *P < 0.05.

To understand the role of MKP-2 in EAE, we first examined whether there is a correlation between EAE disease severity and the expression levels of MKP-2. C57BL/6 mice were immunised, spinal cord, spleen and dLN tissues of EAE mice were collected at the stages of disease onset (day 9–11, when mice develop tail paralysis), peak (day 15–17 when mice exhibit hind limb paralysis) and resolution (day 26–28 when mice had steady reduced clinical scores after the peak). The expression of MKP-2 mRNA was measured by qPCR and our data show that there was a significant 1.9 ± 0.2-fold increase in the mRNA of MKP-2 in EAE spinal cord tissues at disease onset, and the levels were further increased to 3.8 ± 0.8-fold at EAE peak, when compared with spinal cord tissues of naïve controls (Fig. 1A). At the resolution stage, when mice display milder clinical symptoms of EAE, MKP-2 mRNA levels in the spinal cord were comparable between EAE and naïve counterparts.

To study whether MKP-2 is also a potential mediator of the peripheral immune responses in CNS inflammatory disease, we next determined the expression levels of MKP-2 in the spleen and dLN tissues during EAE development. Our data show that there was a significant increase of MKP-2 mRNA expression in both spleen and dLN tissues in EAE mice compared to naïve animals at peak and resolution stages (Fig. 1B,C), with MKP-2 also significantly upregulated in dLN tissues at EAE onset. We further assessed antigen specific MKP-2 expression in EAE spleen and dLN cells by *ex vivo* MOG peptide stimulation. Single cell suspensions were cultured with or without MOG_35–55_ for up to 4 hours before cells were collected and MKP-2 expression analysed by qPCR. Our results show that in spleen cells (Fig. 1D), MKP-2 mRNA expression was significantly increased by 5.8 ± 1.1-fold relative to unstimulated cells after 1 hour (Fig. 1D), and levels began to decrease after this but remained significantly upregulated. The expression levels were also significantly increased in MOG_35-55_ cultured LN cells at 2 and 4 hours, with the expression at 3.4 ± 0.6-fold and 4.7 ± 1.3-fold higher respectively (Fig. 1E).

### MKP-2 deficient mice are less susceptible to EAE

**Figure 2:**
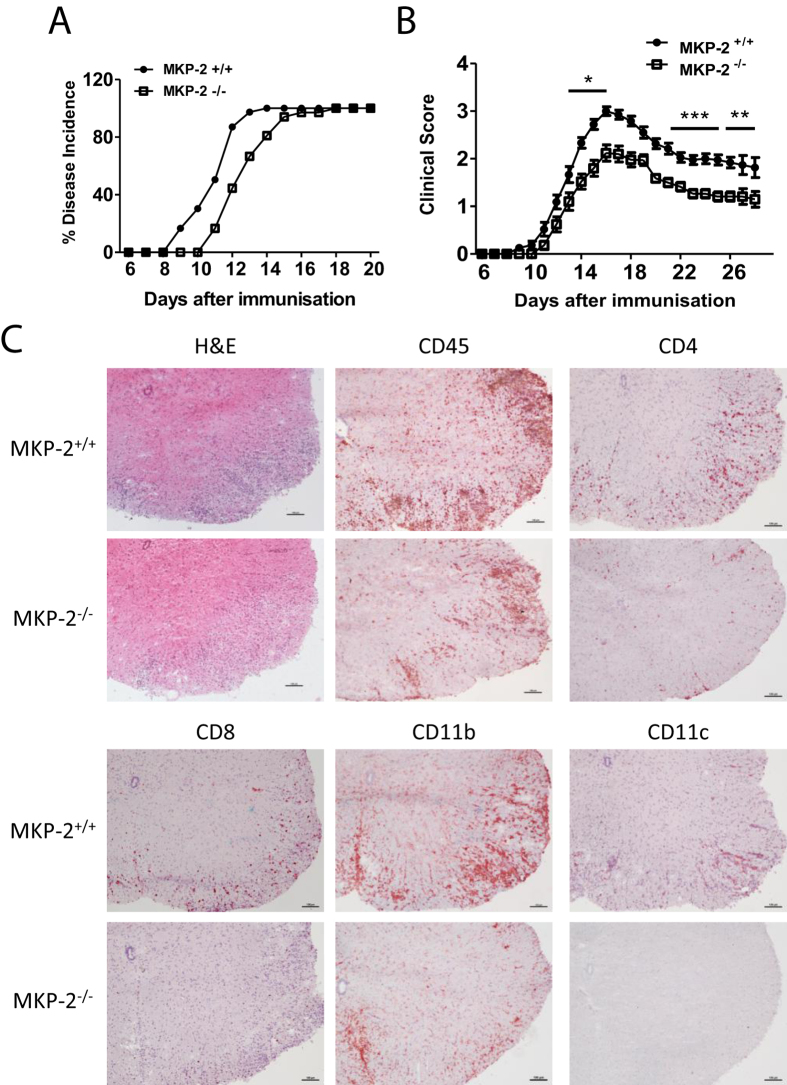
MKP-2^−/−^ mice develop less severe EAE then MKP-2^+/+^ counterparts. MKP-2^+/+^ and MKP-2^−/−^ mice were inmmunised as described in Materials and Methods. (**A**) EAE incidence in MKP-2^+/+^ and MKP-2^−/−^ mice, n = 24 in each group. (**B**) Clinical score of EAE development in MKP-2^+/+^ and MKP-2^−/−^ mice. Data show mean ± SEM of 24 mice per group from at least 4 independent experiments. *P < 0.05; **P < 0.01, ***P < 0.001. (**C**) H&E, CD45, CD4, CD8, CD11b and CD11c staining of spinal cords 17 days post immunisation. Images are representative of at least 12 mice per group from three independent experiments, scale bars = 100 μm.

In order to determine how prominent MKP-2 is in EAE pathogenesis, we investigated the effect of *mkp-2* gene disruption on disease development and progression. EAE was induced in MKP-2^−/−^ mice and MKP-2^+/+^ littermates. Our data show that there was no difference in the overall incidence of disease between the two groups as all mice in both groups developed EAE (Fig. 2A). However, while MKP-2^+/+^ mice started to show EAE signs at day 9 and all mice developed EAE at day 14 after immunisation, MKP-2^−/−^ mice had a delayed disease onset showing loss of tail tone at day 11 and not reaching 100% incidence until day 18 (Fig. 2A). Furthermore, MKP-2^−/−^ mice developed significantly less severe clinical symptoms of EAE compared to MKP-2^+/+^ mice throughout the time course (Fig. 2B), with the average EAE score of the MKP-2^+/+^ group reaching a peak of 3 compared to just 2.1 in MKP-2^−/−^ mice.

To investigate the extent of inflammation and cellular infiltration in the CNS, spinal cord tissues from peak EAE mice were harvested and H&E staining was carried out. Confirming the clinical data that less severe EAE was observed in MKP2^−/−^ mice, histological examination showed reduced lesions and reduced number of infiltrating cells particularly in the white matter of MKP-2^−/−^ spinal cord sections compared to MKP-2^+/+^ tissue (Fig. 2C). Following that, we performed immunohistochemical staining of specific immune cell surface markers to identify the CNS infiltrating cell phenotype. Data in Fig. 2C demonstrate that there was a dramatic decrease of CD45^+^ infiltrating immune cells in the lesions of MKP-2^−/−^ mice spinal cords compared with MKP-2^+/+^ tissues, and that the majority of these infiltrating cells were likely to be CD4^+^ T cells and CD11b^+^ macrophages, together with some CD8^+^ and CD11c^+^ cells. These data thus suggest that MKP-2 deficiency leads to reduced CNS inflammation.

### MKP-2 deficient EAE mice had altered antigen specific cytokine production profile

**Figure 3:**
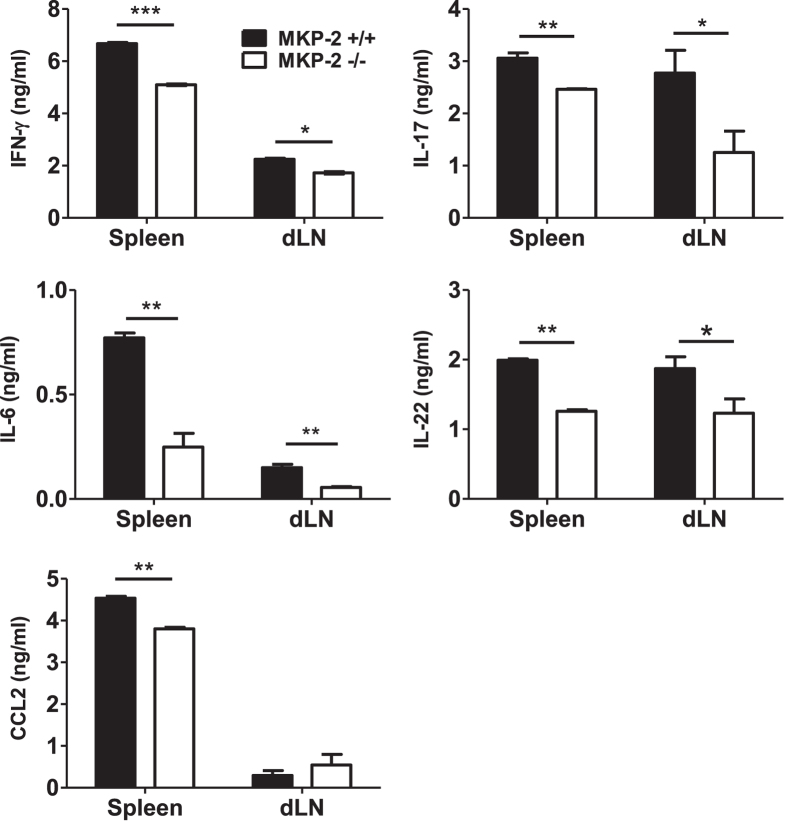
MKP-2^−/−^ mice display a reduced immune response during EAE development compared to MKP-2^+/+^ mice. Spleens and dLNs were harvested at EAE peak and disrupted to form individual cell suspensions. Cells were stimulated with or without MOG_35-55_ for 72 hours and supernatants collected for analysis of cytokine expression by ELISA. Graphs show mean ± SEM of three independent experiments, n = 12 per group *P < 0.05; **P < 0.01, ***P < 0.001.

To understand the mechanistic basis of reduced EAE observed in MKP-2^−/−^ mice, we first investigated the cytokine production profile of the immune cells within secondary lymphoid tissues during EAE progression. Spleen and dLNs cells were collected from MOG peptide immunized MKP-2^+/+^ and MKP-2^−/−^ mice at EAE peak stage. Single cell suspensions were collected and cultured for 72 hours with or without MOG_35-55_ and supernatants collected for analysis of various cytokines by ELISA. While very low levels of cytokine and chemokine were detected in the supernatants of cultures without MOG_35-55_ and with no difference between the two groups (data not shown), our results show that there was a MOG-specific response induced in both groups of lymphoid cells. The spleen and dLN cells of MKP-2^−/−^ mice produced significantly less antigen specific IFN-γ, IL-17, IL-6 and IL-22 in the culture compared to MKP-2^+/+^ (Fig. 3), and the difference was more significant in spleen tissues. In addition, production of the chemokine CCL-2 was also significantly reduced in MKP-2^−/−^ spleen cells (Fig. 3).

### MKP-2 deficient EAE mice had altered immune cell phenotype

**Figure 4:**
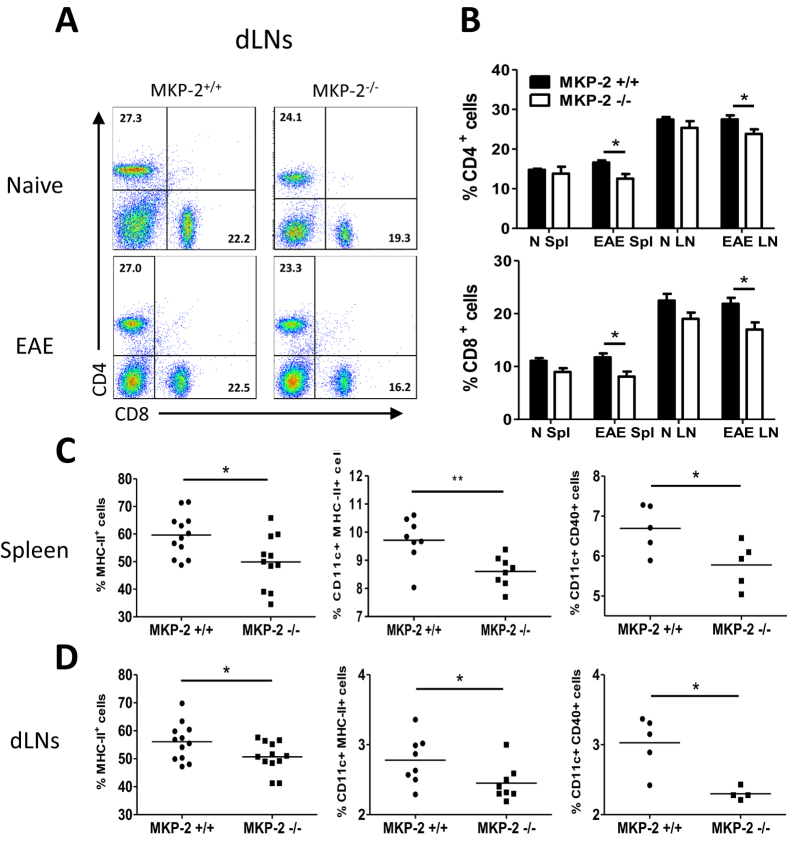
Altered phenotypte of immune cells in naïve and EAE MKP-2^+/+^ and MKP-2^−/−^ mice lymphoid organs. (**A** and **B**) Cells from naïve and EAE spleen and dLNs were stained for CD4 and CD8 and analysed by flow cytometry. (**A**) FACS plots of dLN tissue cells are representative of at least 10 mice per EAE group (MKP-2^+/+^ and MKP-2^−/−^ mice) from three independent experiments. (**B**) Bar graphs show combined data from three independent experiments with n = 10 in each group, N refers to naïve, and Spl refers to spleen in the x-axis labelling, bars represent mean ± SEM. (**C** and **D**) Expression of MHC-II, CD11c and CD40 by MKP-2^+/+^ and MKP-2^−/−^ immune cells in EAE spleen (**C**) and dLNs (**D**). Spleen and dLNs were harvested at EAE peak and disrupted to form individual cell suspensions. Cells were then stained and analysed for expression of MHC-II, or expression of both CD11c and MHC-II molecules, or both CD11c and CD40 by flow cytometry. Graphs show combined data from three independent experiments, each symbol represents an individual mouse and bar indicates the average value of expression in each group. *P < 0.05; **P < 0.01.

Next we analysed the immune cell phenotype in the spleen and dLN tissues of MKP-2^+/+^ and MKP-2^−/−^ naïve and EAE mice (Fig. 4). Single cell suspensions were collected and stained for various cell surface markers and analysed by flow cytometry. Expression of CD4 and CD8 T cells were first examined (Fig. 4A and B). Our data show that no difference was observed in the frequency of CD4 and CD8 positive cells in the lymphoid tissues between naïve MKP-2^+/+^ and MKP-2^−/−^ mice. However, in EAE mice, the frequency of CD4^+^ T cells and CD8^+^ T cells was significantly decreased in the MKP-2^−/−^ group compared with MKP-2^+/+^ littermates at day 17 after immunisation with peak EAE clinical severity.

Further analysis of myeloid cell populations indicated that there was no difference of CD11c and CD11b expression in the spleen or dLNs tissues between the WT and KO mice in naïve or EAE immunised mice (data not shown). However there was a significant reduction in the frequency of MHC-II^+^ cells in the spleen (Fig. 4C) and dLNs (Fig. 4D) tissues of MKP-2^−/−^ mice when compared to that of the MKP-2^+/+^ littermates at the peak of EAE, whereas no such difference was observed between naïve MKP-2^−/−^ and MKP-2^+/+^ mice (data not shown). As DCs are professional antigen presenting cells and activated DCs express high levels of MHC-II, we studied the frequency of CD11c^+^ MHC-II^+^ cells in spleen and LNs. We observed a significant reduction in the frequency of CD11c and MHC-II double positive cells in the lymphoid organs of MKP-2^−/−^ EAE mice compared with MKP-2^+/+^ controls (Fig. 4C and D). In addition, the percentage of CD11c^+^ CD40^+^ cells was also significantly reduced in the MKP-2^−/−^ mice (Fig. 4C and D). The reduced EAE severity in MKP-2 deficient mice thus was likely associated with reduced frequency of lymphocyte numbers, as well as MHC-II^+^ and CD40^+^ DCs in the spleen and dLNs.

### MKP-2 deletion impairs CD4^+^ T cell activation

**Figure 5:**
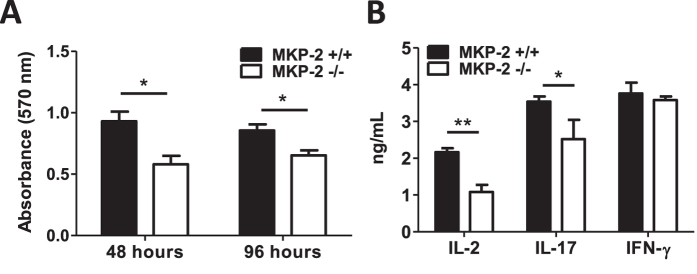
CD4^+^ T cells of non-immunised MKP-2^−/−^ mice have reduced cell proliferation and cytokine production. CD4^+^ T cells were isolated from spleens of MKP-2^+/+^ and MKP-2^−/−^ mice, and then activated in culture with anti-CD3 and anti-CD28 antibodies. (**A**) After 48 and 96 hours, T cell proliferation was measured by MTT assay. (**B**) Cell supernatants were also collected after 72 hours of culture and then cytokine levels were measured by ELISA. Values represent mean±SEM of three independent experiments, *P < 0.05; **p < 0.01.

In order to determine whether the altered immune cell cytokine production and reduced T cell frequency in EAE development may be due to an intrinsic change in T cell function as a result of MKP-2 deletion, CD4^+^ T cells were purified from spleens of naïve MKP-2^+/+^ and MKP-2^−/−^ mice and activated with plate-bound anti-CD3 and anti-CD28. T cell proliferation was measured by MTT assay after 48 hours and 96 hours of culture, and cytokine levels in the supernatants after 72 hours culture were measured by ELISA. Our data show that MKP-2^−/−^ T cells display reduced proliferation following the polyclonal activation compared to MKP-2^+/+^ cells (Fig. 5A). In addition, CD4^+^ T cells from MKP-2^−/−^ mice produced less IL-2 and IL-17 whereas IFN-γ production was unchanged between the two groups (Fig. 5B). These data suggests that MKP-2 is involved in regulation of CD4^+^ T cell proliferation but may play a more important role in specific CD4^+^ T cell subsets such as Th17 cells.

### MKP-2 deletion alters DC phenotype and function

**Figure 6:**
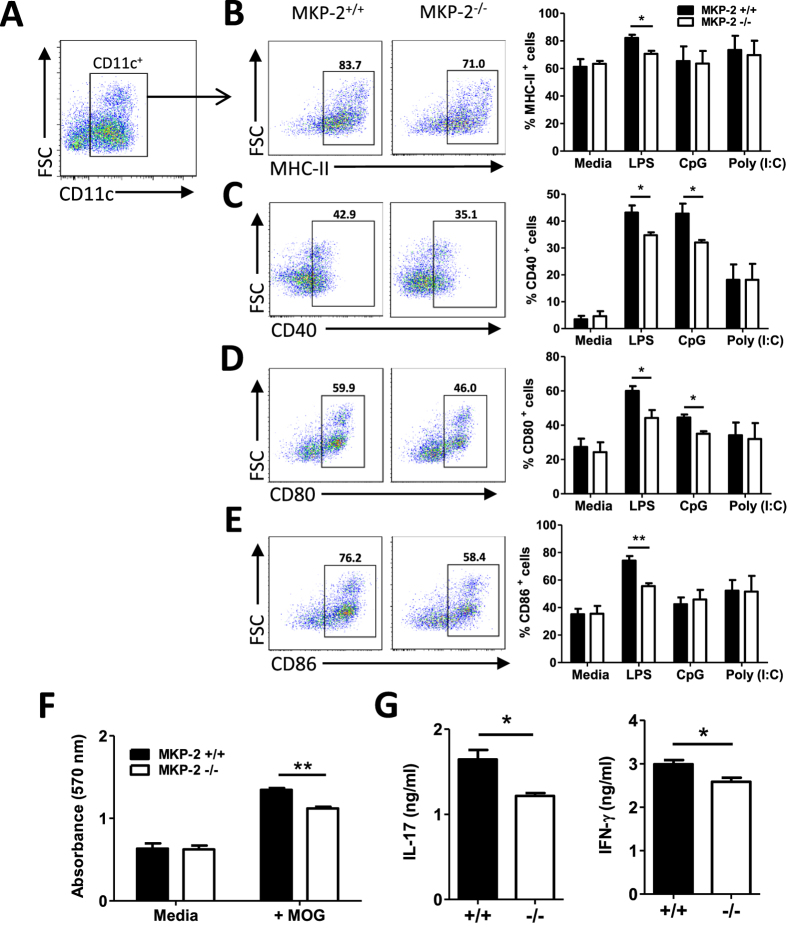
BMDCs of MKP ^−/−^ mice exhibit reduced expression of MHC-II and costimulatory molecules. BMDCs were generated from the culture of bone marrow from MKP-2^+/+^ and MKP-2^−/−^ mice. Cells were stimulated with or without LPS (100ng/ml), CpG (1 μM) or Poly(I:C) (20 μg/ml) for 24 hours and CD11c, MHC-II, CD40, CD80 and CD86 expression analysed by flow cytometry. FACS plots are representative of three independent experiments of BMDCs activated by LPS. (**A**) Cells were gated for CD11c^+^ cells first, and then the gated positive cells were analysed for their expression of (**B**) MHC-II, (**C**) CD40, (**D**) CD80 and (**E**) CD86. (**B–E**) Graphs show combined data from three independent experiments, bars represent mean ± SEM. (**F** and **G**) BMDCs from MKP-2^+/+^ and MKP-2^−/−^ mice were pulsed with or without MOG_35-55_ (100 μg/ml) for 4 hours before incubation with purified CD4^+^ T cells isolated from wild type EAE mice spleen and dLN tissues. (**F**) After 48 hours cell proliferation was determined by MTT assay. (**G**) After 72 hours of culture, cell supernatants were collected for cytokine production analysis using ELISA. Values represent mean ± SEM of two independent experiments performed in triplicate. *P < 0.05; **P < 0.01, ***P < 0.001.

To further investigate whether the phenotypically altered DCs may also contribute to the decreased proinflammatory response, and thus attenuated EAE severity, in MKP-2^−/−^ mice, we next examined the phenotype and function of DCs derived from the bone marrow of MKP-2^−/−^ and MKP-2^+/+^ mice. BMDCs were generated in culture with GM-CSF and cells were then stimulated with various TLR ligands: 100 ng/ml LPS, 1 μM CpG or 20 μg/ml Poly(I:C) for 24 hours before being analysed for the expression of CD11c, MHC-II, CD80, CD86 and CD40 by flow cytometry (Fig. 6A–E). Our data shows that about 54% of MKP-2^−/−^ CD11c^+^ BMDCs cultured in media alone were MHC-II^+^, significantly less than MKP-2^+/+^ cells with 62% (Fig. 6B). Following LPS stimulation, the proportion of MHC-II^+^ CD11c^+^ cells from MKP-2^+/+^ mice was 71% with a significant increase of 9%. In contrast, activated MKP-2^−/−^ BMDCs displayed only a small 3% increase in MHC-II expression (Fig. 6B). Therefore our data show that MKP-2^−/−^ BMDCs express reduced levels of MHC-II relative to their MKP-2^+/+^ counterparts and also are less capable of upregulating MHC-II in response to immune challenge such as TLR4 ligands. Similarly, expression of the co-stimulatory molecules CD40 (Fig. 6C), CD80 (Fig. 6D) and CD86 (Fig. 6E), were also significantly lower in MKP-2^−/−^ mice derived BMDCs compared to the cells of MKP-2^+/+^ mice following LPS stimulation, with CD80 and CD86 levels reduced on unstimulated cells similar to MHC-II. It is worth noting that CD40 and CD80 expression was also reduced in MKP-2^−/−^ BMDCs in response to TLR9 ligand CpG activation, but not Poly(I:C), a TLR3 agonist (Fig. 6), suggesting MKP-2 affects TLR4 and TLR9 pathways but not TLR3 in BMDCs.

To further test whether the reduced levels of MHC-II and costimulatory molecules had any functional impact, the antigen presentation/T cell activation capability of the BMDCs was examined. BMDCs derived from naïve MKP-2^+/+^ and MKP-2^−/−^ mice were pulsed with or without MOG_35-55_ peptide, cells were then washed thoroughly and cocultured with CD4^+^ T cells isolated from wild type peak EAE mice followed by analysis of T cell proliferation and cytokine production. As expected, T cells co-cultured with MOG_35-55_ pulsed BMDCs (+MOG) had significantly increased levels of proliferation compared with BMDCs without MOG (Medium) stimulation before co-culture (Fig. 6F). However those T cells co-cultured with MOG peptide pulsed BMDCs of MKP-2^−/−^ mice had reduced proliferation compared to cells cultured with MKP-2^+/+^ BMDCs (Fig. 6F). Similarly, while co-culture of T cells with medium pulsed BMDCs had undetectable levels of cytokine production (data not shown), T cells co-cultured with MOG peptide pulsed BMDCs produced high levels of IL-17 and IFN-γ, and the levels were significantly reduced in the co-culture supernatants of MKP-2^−/−^ mice BMDCs compared with DCs from MKP-2^+/+^ mice (Fig. 6G).

### MKP-2 deletion alters macrophage phenotype and function

**Figure 7:**
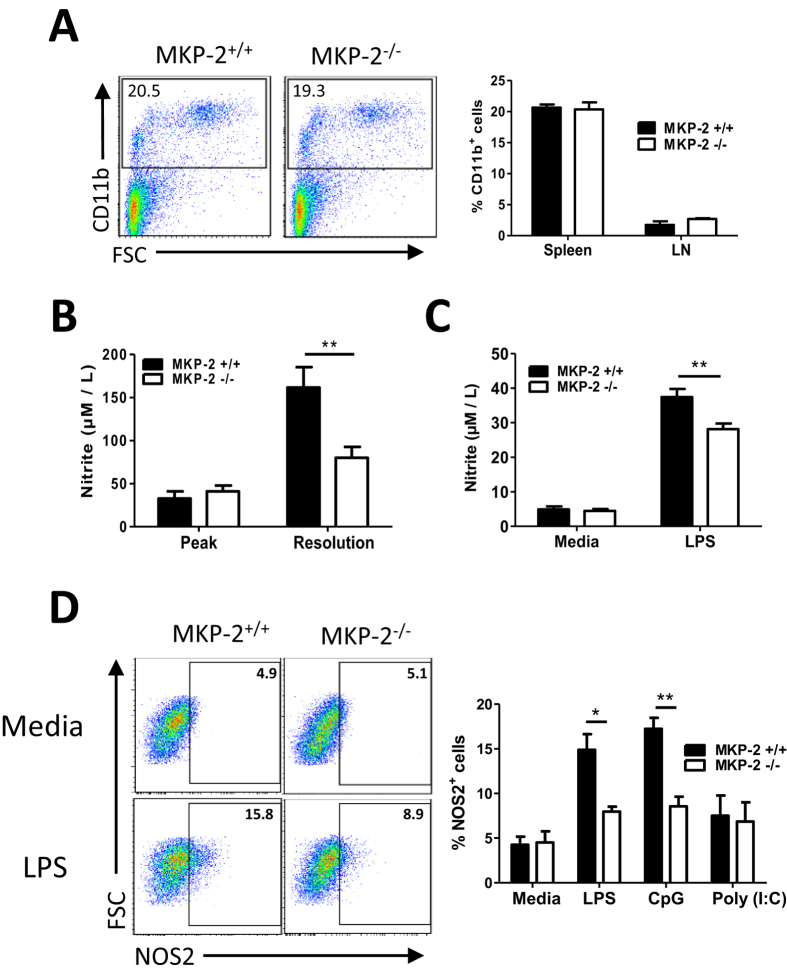
MKP-2 is important for NO production by macrophages. (**A**) Spleen and dLNs were harvested at EAE peak and disrupted to form individual cell suspensions and cells stained for CD11b expression. FACS plots are representative of at least 9 mice per group from three independent experiments. Graphs show combined data from three independent experiments, bars represent mean ± SEM. (**B**) Whole blood was harvested at EAE peak and resolution stages and nitrite concentrations determined by Griess Assay. Graphs show mean ± SEM of two independent experiments (n = 7 MKP-2^+/+^; n = 8 MKP-2^−/−^). (**C**) BMMs were generated from the bone marrow from MKP-2^+/+^ and MKP-2^−/−^ mice. Cells were stimulated with or without LPS (100 ng/ml) and culture supernatant was collected after 24 hours and nitrite concentrations determined by Griess Assay. Values represent mean ± SEM of three independent experiments. (**D**) BMMs were stimulated with or without LPS (100 ng/ml), CpG (1 μM) or Poly(I:C) (20 μg/ml) for 24 hours and NOS2 expression was analysed by flow cytometry following initial gating on CD11b^+^ cells. FACS plots are representative of three independent experiments of BMMs either unstimulated or activated by LPS. Graphs show combined data from three independent experiments, bars represent mean ± SEM. *P < 0.05, **P < 0.01.

The role of MKP-2 in infection has been closely linked with its effect on macrophage cell activation including cytokine release and NO production^22^,^24^. We did not observe any difference in the frequency of CD11b^+^ cells in the spleen and dLNs tissues between MKP-2 WT and KO littermates in naïve (data not shown) and EAE conditions (Fig. 7A). When we analysed the NO formation in the serum samples of MKP-2^+/+^ and MKP-2^−/−^ EAE mice using a standard Griess reagent system, our results show that nitrite levels were significantly increased in EAE mice compared to naïve controls as expected (data now shown). However to our surprise there was no difference in NO levels between the serum samples of MKP-2^+/+^ and MKP-2^−/−^ at EAE peak stage, only at the resolution stage we observed a significant reduction of nitrite, decreasing from 161.8 ± 21.9 μM in the MKP-2^+/+^ mice to 80.1 ± 33.8 μM in the MKP-2 deficient mice (Fig. 7B). We also examined NO production by BMMs generated from MKP-2^+/+^ and MKP-2^−/−^ mice *in vitro*. Our results indicate that while unstimulated MKP-2^+/+^ and MKP-2^−/−^ BMMs produced low levels of nitrite with no difference between the two groups (Fig. 7C), nitrite concentrations were increased upon LPS stimulation, and the level was consistently significantly reduced in MKP-2^−/−^ BMMs compared to MKP-2^+/+^ counterparts (Fig. 7C), confirming the importance of MKP-2 in macrophage activation and function. Interestingly similar to BMDCs, MKP-2 deletion affected macrophage activation in response to both LPS and CpG, but not Poly (I:C), as the frequency of NOS2 + cells were significantly increased in the MKP-2^+/+^ BMMs after LPS and CpG stimulation (Fig. 7D).

## Discussion

The function of MKP-2 in MS disease development is of particular interest as MKP-2 is widely expressed in the brain tissues^25^ in addition to its function in the immune system, and MS is a disease characterised by immune mediated inflammation in CNS. In this report we have shown that MKP-2 expression is significantly enhanced in the spinal cord as well as the peripheral spleen and dLNs at the onset and peak of EAE, suggesting MKP-2 is involved in the initiation and development of EAE possibly through mediating the immune responses in the peripheral lymphoid organs as well as within the CNS. Indeed, MKP-2 deficient mice developed attenuated EAE, which was shown with reduced CNS inflammation and decreased number of infiltrating immune cells. Peripheral immune organs exhibited a reduced percentage of CD4^+^ and CD8^+^ T cells and reduced antigen specific proinflammatory cytokine production, and this was likely due to the impaired function of T cells and antigen presenting DCs. This study therefore suggests that MKP-2 plays an important role in the development of CNS inflammatory diseases possibly through modulating the function of key antigen presenting cells and T cells.

Despite recent effort to understand the roles of MKPs in immune regulation, it is currently not known whether MKP-2 plays a role in the development of neuroinflammation in CNS. Our findings of attenuated EAE in the MKP-2^−/−^ mice together with significantly reduced percentages of CD4^+^ and CD8^+^ cells in the spleen and dLNs compared to MKP-2^+/+^ mice indicate that MKP-2 is likely to be essential for CD4^+^ and CD8^+^ T cell expansion in lymphoid organs during the development of CNS inflammation. Furthermore, CD4^+^ T cells from naïve MKP-2^−/−^ mice were shown to have reduced cell proliferation and impaired IL-17 and IL-2 production in response to CD3/CD28 activation, suggesting an intrinsic functional alteration of these T cells due to MKP-2 deletion. The data agree with our previous reports that MKP-2 deficient mice had a diminished Th1 response or a general T cell hypo-responsiveness depending on the infected pathogens^22^,^24^. Interestingly while we observed an impaired IL-17 and IL-2 production by T cells from MKP deficient mice, IFN-γ production was comparable to that of MKP-2^+/+^ mice, suggesting that MKP-2 is likely to be involved in the activation of specific T cells and immune molecules. Nevertheless, our study supports the recent findings from other groups^1^,^15^,^26^,^27^ that MKPs are critical regulators of T cell activation and function. For example, MKP-1 was shown to be required for T cell activation and function. T cells deficient in MKP-1 had impaired cell activation, proliferation, and function *in vitro* and the gene deficient mice were more resistant to EAE development^15^. MKP-5 has differential roles in the regulation of T cell clonal expansion and effector T cell cytokine expression, MKP-5 deficient T cells proliferated poorly upon activation which resulted in increased resistance to EAE than the wild type controls^16^. The emerging research evidence thus suggests that MKPs play important roles in the regulation of inflammatory responses such as sepsis, infection and CNS inflammation^2^,^3^,^5^,^21^, and they may become potential therapeutic targets for inflammatory diseases such as MS.

Associated with the reduced EAE severity and T cell expansion in the MKP-2^−/−^ mice, our data also indicate that the levels of antigen specific IL-6, IL-17, IL-22 and IFN-γ production by *ex vivo* cultured lymphoid cells from both spleen and dLNs were all significantly reduced in the MKP-2^−/−^ group compared to MKP-2^+/+^ controls. Therefore a further possible mechanism of action of MKP-2 during EAE pathogenesis and progression is via the regulation of cytokine production, including IL-17 and IFN-γ which are secreted by Th17 and Th1 cells respectively. While several previous studies suggest MKPs such as MKP-1 can dynamically regulate both pro- and anti-inflammatory cytokine production e.g. IL-6, TNF-α and IL-10 by innate immune cells^8^,^9^,^10^, two very recent reports suggest that some phosphatases are able to directly regulate Th17 cell differentiation and function. MKP-1 is shown to control IL-17R signalling and tissue inflammation by the p38α-MKP-1 signalling axis in EAE^28^, while DUSP2 regulates Th17 differentiation through controlling the activity of STAT3^29^. Our findings of reduced levels of IL-17 by naïve MKP-2^−/−^ mice CD4^+^ T cells in response to CD3/CD28 activation, together with decreased antigen specific production of IL-17 and IL-22, a cytokine that can also be produced by Th17 cells along with IL-17, by cultured spleen and dLNs cells of MKP-2^−/−^ mice support a potential role of MKP-2 in regulating Th17 cells, but the underpinning molecular mechanisms remain to be determined.

It is not clear what caused the dramatically reduced inflammatory cell infiltration into the spinal cord in the MKP-2 deficient mice, one possible explanation could be the altered chemokine expression by immune cells in the absence of MKP-2 which affects cell migration to areas of inflammation. Reduction of neutrophil infiltration in the lung of MKP-2^−/−^ mice was closely associated with decreased MIP-1α expression in BAL fluids in a mouse model of ALI^20^. Furthermore, other MKPs including MKP-1 have also been shown to control chemokine expression such as CCL2, CCL3, CXCL1 and CXCL2 in response to LPS administration^10^,^13^,^30^. In our study we observed that CCL2 level was significantly decreased in MKP-2^−/−^ EAE spleen cells following MOG stimulation. As CCL2 acts as a chemoattractant for a variety of immune cells, MKP-2 could potentially affect the level of cell migration to sites of inflammation, thereby increasing autoimmune pathology.

MKP-2 is shown to exhibit important functions in modulating immune responses, particularly T cells, in EAE and various diseases as shown by us and others^20^,^21^,^22^,^23^,^24^. However it is not yet known whether and how it regulates the key antigen presenting DCs. We observed a significantly reduced frequency of CD11c^+^ MHC-II^+^ DCs in MKP-2 deficient mice spleen and dLNs at peak of EAE disease compared with MKP-2^+/+^ controls. Similar observations were also seen in BMDCs, with MKP-2^−/−^ mice cells expressing significantly lower levels of MHC-II as well as the costimulatory molecules CD40, CD80 and CD86 in response to LPS. While MKP-2 deletion altered BMDC activation by LPS and CpG, ligands of TLR4 and TLR9 respectively, it did not affect the signalling via TLR3, suggesting MKP-2’s function varies in different TLR signalling pathways and this was also confirmed in BMM cells. Furthermore, functional tests of BMDCs co-cultured with purified CD4^+^ T cells from wild type EAE mice indicate the BMDCs of MKP-2^−/−^ mice had impaired capability of activating antigen specific T cells as the co-cultured cells had reduced proliferation and IL-17 and IFN-γ production. Our data therefore suggest an important but currently unrecognised role of MKP-2 in directly regulating DC function. In addition to the intrinsic defect of CD4^+^ T cells, MKP-2 deficiency alters DC activation phenotype which results in impaired antigen presentation and reduced T cell function and cytokine production, and ultimately decreases clinical symptoms in EAE. It is not yet clear how MKP-2 modulates MHC-II expression in DCs. Previous reports suggest MHC-II gene expression is regulated by the transcriptional coactivators RFX and CIITA^31^,^32^, while the expression of CIITA is controlled by ERK and p38 activity through a negative feedback loop^33^. Indeed MKP-1 deficient macrophages and DCs displayed reduced expression of CIITA and MHC-II after being activated^33^. Therefore it would be interesting to know whether the reduced expression of MHC-II in MKP-2^−/−^ DCs is due to altered CIITA expression via MAPK dephosphorylation.

Whilst our data here suggest a novel function of MKP-2 in regulating DC function and T cell activation, the importance of MKP-2 in macrophage differentiation and activation has been well documented, with MKP-2 deficiency potentiating LPS-induced production of inflammatory cytokines IL-6, IL-12 and TNF-α by BMMs, as well as modulating iNOS and Arginase-1 production^22^,^23^. In our study we observed similar differences in BMM phenotype and cytokine production profile between the MKP-2^+/+^ and MKP-2^−/−^ BMMs after LPS stimulation (data not shown), confirming these previous reports. In addition, our data revealed that BMMs from MKP-2^−/−^ mice had reduced levels of NO production after LPS activation, and this was confirmed in EAE mice at day 28 after immunisation. While the positive regulation of MKP-2 on NO production would enhance disease pathogenesis as NO is known to contribute to the inflammatory response and demyelination of MS/EAE, the difference of NO levels only shown on day 28 but not day 17 after immunisation may suggest a dominant role of MKP-2 in modulating the adaptive immune response in the development of EAE through its effect on antigen presenting DC function, rather than macrophages. We also observed no difference in the production of reactive oxygen species by PMA activated neutrophils purified from MKP-2^+/+^ and MKP-2^−/−^ mice bone marrow tissues (data not shown), further confirming a specific role of MKP-2 in myeloid DC activation and function.

While our study focused on the role of MKP-2 in the immune response during EAE development, it is worth noting that various MKPs are important in CNS function and diseases. MKP-3 regulates the activities of oligodendrocytes, microglia and neurons^34^,^35^. MKP-2 is constitutively expressed by neuronal cells and regulates neuronal differentiation^25^. In our study, we observed the constitutive expression of MKP-2 in the murine spinal cord tissues, and furthermore its expression was significantly increased in EAE mice at the disease onset and peak stages compared with naïve mouse tissues, suggesting a potential role of MKP-2 in CNS compartment. Future experiments identifying MKP-2 expressing cells in both the CNS and immune systems would lead to better understanding of the specific function of MKP-2 in diseases such as MS. While our further analysis underlines how MKP-2 modulates the peripheral immune responses, it is not known whether the molecule might also influence EAE development and recovery through modulating CNS resident cells, e.g. myeloid microglia cells, and therefore CNS function such as neuron damage, repair, or demyelination/remyelination, and subsequently contribute to disease clinical symptoms. Future studies determining the function of MKP-2 in the CNS will increase our understanding of its role in MS development.

In conclusion, our study reveals the important role of MKP-2 in EAE. MKP-2 deletion results in attenuated EAE severity, which is associated with reduced production of inflammatory cytokines by lymphocytes. And the potential mechanism of MKP-2 action in EAE is likely to be via antigen presenting DCs, as MKP-2 is required for the upregulation of MHC-II and costimulatory molecules during DC activation, and is essential in T cell activation and function. The data suggest that inhibition of MKP-2 expression or function may be a viable strategy in the treatment of CNS inflammatory diseases such as MS.

## Materials and Methods

### Mice

Naïve C57BL/6 mice, and MKP-2^+/+^ and MKP-2^−/−^ littermates on a C57BL/6 background were bred and maintained at the Biological Procedure Unit at the University of Strathclyde. MKP-2^−/−^ mice were originally generated by Professor Plevin as previously described^22^. Female animals at 7–8 weeks of age were used in all experiments. All animal experiments were performed under the guidelines of the UK Animals (Scientific Procedures) Act 1986 and were conducted under a Project License granted by the UK Home Office and approved by the University of Strathclyde’s Animal Welfare and Ethical Review Body.

### EAE induction and Assessment

Naïve C57BL/6 mice or MKP-2^−/−^ and MKP-2^+/+^ littermates were immunized subcutaneously on day 0 with 100 μl of 100 μg MOG_35–55_ (ChinaPeptides Co Ltd) emulsified in complete freunds adjuvant (CFA; Sigma) supplemented with 3.65 mg Mycobacterium *tuberculosis* (BD Biosciences). In addition, each mouse received 100 ng pertussis toxin (PTX; Tocris Bioscience) in 100 μl PBS injected intraperitoneally on day 0 and again on day 2. Mice were monitored daily for signs of disease development and given a clinical score based on the following evaluation system: 0 = no clinical sign; 0.5 = partial loss of tail tone; 1.0 = complete loss of tail tone; 1.5 = altered gait; 2.0 = hind limb weakness; 2.5 = paralysis of one leg; 3.0 = hind limb paralysis; 3.5 = hind limb paralysis with significantly reduced mobility; 4.0 = forelimb involvement; 5.0 = moribund.

### Spinal cord collection and histology

MKP-2^+/+^ and MKP-2^−/−^ EAE mice were sacrificed at the planned dates. Following PBS perfusion, intact spinal cords were flushed out with PBS by hydrostatic pressure using a sterile 19G needle. Frozen spinal cord tissue sections were then stained with standard haematoxylin and eosin (H&E) staining or with anti-CD45, anti-CD4, anti-CD8, anti-CD11b and anti-CD11c antibodies (all purchased from eBioscience) followed by incubation with an appropriate biotin-conjugated secondary antibody (eBioscience, UK), horseradish *peroxidise* and ImmPACT AMEC red peroxidase substrate (Vector Lab, UK) for detection. Isotypes with matching IgG were used as negative controls for all antibody staining.

### Quantitative PCR

Mice were sacrificed at various time points and spinal cord, spleen and inguinal draining lymph nodes (dLNs) were collected. RNA isolation was performed using TRIzol reagent (Life Technologies) as per manufacturer’s guidelines. Next, 2 μg total RNA was reverse transcribed using the High Capacity cDNA Reverse Transcription Kit (Life Technologies UK). The cDNA obtained from reverse transcription was analysed for expression of various mRNA transcripts by quantitative PCR (qPCR) using the 2x Fast SYBR Green master mix (Life Technologies). MKP-2 and β-actin Primers were obtained from Sigma-Aldrich and designed using Primer3 software. List of primers used, (5′–3′): MKP-2 forward TGCCAGAGAAGACTTGGTTT, MKP-2 reverse TCCTCTTTCCTTTCCCTCTC; β-actin forward GTGGGCCGCTCTAGGCACCAA, β-actin reverse CTCTTTGATGTCACGCACGATTTC.

### Spleen and draining lymph node cell culture

Mice were sacrificed at various time points and spleen and dLNs were harvested for cell culture. Single cell suspensions from each mouse were cultured in 24-well plates at 4 × 10^6^ cells/2 ml per well for spleen or 2 × 10^6^ cells/2 ml per well for dLNs and stimulated with or without 40 μg/ml MOG_35–55_. For mRNA analysis, cells were collected at 10 mins, 30 mins, 1 hour, 2 hours and 4 hours after MOG stimulation and MKP-2 mRNA levels were determined by qPCR. For cytokine analysis, supernatants from the culture were collected after 72 hours and the concentrations of IL-17, IFN-γ, IL-6, IL-22and CCL2 were determined by ELISA. All ELISA kits were purchased from eBioscience.

For polyclonal activation of CD4^+^ T cells, spleens from both MKP-2^−/−^ and MKP-2^+/+^ mice were collected and cells were purified from single cell suspensions using the CD4^+^ T cell isolation kit (Miltenyi Biotec) per manufacturers guidelines. Following this, purified CD4^+^ T cells (2 × 10^5^) were added to 96-well plates which had been pre-coated overnight with 5 μg/ml anti-CD3e (eBioscience). Soluble anti-CD28 (2 μg/ml; eBioscience) was then added to each well and the cells incubated for 48 and 96 hours for cell proliferation MTT assay. Some plates were cultured for 72 hours and supernatants were collected for cytokine production test using ELISA.

### Generation of mouse bone marrow derived cells

Bone marrow derived cells were generated from the culture of bone marrow from 6–7 week old MKP-2^−/−^ and MKP-2^+/+^ mice. Both tibia and femurs were collected from mice and bone marrow flushed out and disaggregated to form a single cell suspension. For BMDCs, cells were re-suspended in growth medium composed of complete RPMI supplemented with GM-CSF. The BMDCs were harvested on day 7 and stimulated with or without 100 ng/ml LPS, 1 μM CpG or 20 μg/ml Poly(I:C) in 12-well plates (2 × 10^6^ cells/well), cells and supernatants were then harvested for various immunoassays. For bone marrow derived macrophages (BMMs), cells were cultured in complete DMEM growth medium supplemented with 30% L-cell (L929 cell culture derived). After 7 days of culture, BMMs were harvested and stimulated with or without 100 ng/ml LPS, 1 μM CpG or 20 μg/ml Poly(I:C) in 12-well plates (2 × 10^6^ cells/well) for 24 hours and cells and supernatants harvested for immunoassays.

### Flow Cytometry

Individual cells from spleen and dLN tissues or BMDC/BMM culture were added to FACS tubes (0.5 × 10^6^ cells per tube). To block non-specific Fc receptors, cells were resuspended in α-mouse CD16/CD32 Fc block (eBioscience) for 10 minutes before incubation with the appropriate antibodies: anti-CD4, anti-CD8, anti-CD11c, anti-CD40, anti-CD80, anti-CD86, anti-MHC-II, anti-CD11b and anti-NOS2 (all from eBioscience). For intracellular NOS2 analysis, cells were fixed and permeabilized following cell surface staining using the intracellular fixation and permeabilization buffer set (eBioscience). Cells were then resuspended in 0.5 ml FACS buffer and analysed using a BD FACSCanto system and BD FACSDiva software (both BD Biosciences).

### BMDC and T cell co-culture

BMDCs were generated from MKP-2^+/+^ and MKP-2^−/−^ mice. On day 7 cells were harvested and pulsed with or without 100 μg/ml MOG peptide for 4 hours, cells were then washed with PBS thoroughly. Meanwhile spleen and dLNs tissues were collected from MKP-2^+/+^ mice at day 14 after EAE immunisation, and CD4^+^ T cells were isolated by MACS cell separation kit (Miltenyi Biotec). Purified CD4^+^ T cells were then cultured with MOG-pulsed or non-pulsed BMDCs at a ratio of 10:1 (Tc: DC). For analysis of T cell proliferation, cells were cultured in 96-well plates and an MTT assay was performed after 96 hours co-culture. To analyse T cell cytokine production, 24 well plates were used for the co-culture and supernatants were collected after 72 hours and IL-17 and IFN-γ concentrations determined by ELISA.

### Nitric oxide assay

Following euthanasia at the selected time points, blood samples from individual naïve and EAE mouse were harvested and serum collected. For nitric oxide (NO) production by BMMs, culture supernatants were collected after 24 hours LPS incubation. NO levels in the serum and BMMs supernatants were determined by measuring one of its stable oxidation products, nitrite (NO2^−^) by a standard Griess assay using the Griess reagent kit (Molecular Probes; Invitrogen).

### Statistics

All data are shown as mean ± SEM. Statistical analysis of EAE clinical score data was performed using 2-way ANOVA with repeated measures and bonferroni post hoc tests. Other data presented in the study were analysed by two-tailed, unpaired student’s t test, one-way ANOVA or Mann-Whitney U test as appropriate. A *P* value of less than 0.05 was considered statistically significant.

## Additional Information

**How to cite this article**: Barbour, M. *et al*. MAP kinase phosphatase 2 deficient mice develop attenuated experimental autoimmune encephalomyelitis through regulating dendritic cells and T cells. *Sci. Rep.* **6**, 38999; doi: 10.1038/srep38999 (2016).

**Publisher’s note:** Springer Nature remains neutral with regard to jurisdictional claims in published maps and institutional affiliations.
